# The Complete Mitochondrial Genome of the Pest Beetle *Latheticus oryzae*: Assembly, Annotation, and Phylogenetic Analysis

**DOI:** 10.1002/ece3.72717

**Published:** 2025-12-12

**Authors:** Shan Xu, Lihan Cheng, Wennan Hu, Yansong Gao, Qin Cheng, Jun Lv

**Affiliations:** ^1^ School of Food Engineering Moutai Institute Renhuai Guizhou Province China; ^2^ Center for Higher Education Research and Evaluation Moutai Institute Renhuai Guizhou Province China

**Keywords:** adaptive evolution, gene expression, *Latheticus oryzae*, mitogenome, phylogeny, Tenebrionidae

## Abstract

*Latheticus oryzae* (Coleoptera: Tenebrionidae) is a significant pest of stored grains, yet its phylogenetic position and mitogenome characteristics have remained poorly explored. Here, we present the first complete mitochondrial genome (mitogenome) of *L. oryzae* (GenBank accession No. PX331584.1). The complete mitogenome is a circular molecule of 15,798 bp encoding 13 protein‐coding genes (PCGs), 22 tRNAs, 2 rRNAs, and a control region, with a high A + T content of 71.08%. Phylogenetic analysis based on 13 PCGs robustly resolved the evolutionary position of *L. oryzae* , placing it as a sister group to *Tribolium* species within the tribe Tenebrionini. This clarifies its phylogenetic affinities and suggests a close evolutionary relationship and possibly shared adaptive strategies with the model pest genus *Tribolium*. Expression profiling revealed significant developmental and tissue‐specific differences in PCG transcription, with heightened energy metabolism gene expression in larval stages and fat body, potentially reflecting genetic adaptations for growth and survival in nutrient‐rich environments. These findings provide foundational insights into the molecular evolution and ecological adaptation of *L. oryzae* , facilitating future studies on pest evolution and mitogenome‐driven management strategies.

## Graphical abstract

This study presents the first complete mitochondrial genome of the stored grain pest *Latheticus oryzae* , which is 15,798 bp long and exhibits a typical insect mitogenome structure with high A + T content. Phylogenetic analysis confirms its close relationship to Tribolium species within the Tenebrionini tribe. The findings provide a genetic resource for future research on pest management and evolutionary studies.

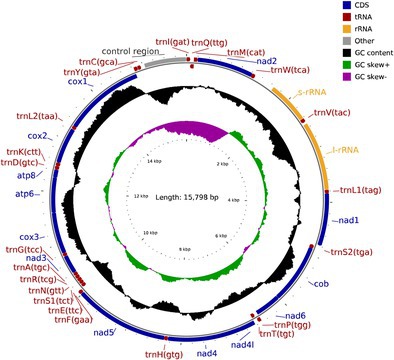

## 1 Introduction

Mitochondrial genomes (mitogenomes) have become pivotal tools in evolutionary biology, offering insights into phylogenetic relationships, population genetics, and adaptive evolution due to their maternal inheritance, high mutation rate, and structural stability (Rebijith et al. 2016; Shao and Barker 2003; Tyagi et al. 2020). In insects, the mitogenome is typically a compact, circular molecule of 14–21 kb, housing 37 genes and a control region that governs replication and transcription (Boore 1999; Cameron 2014). These features make mitogenomes particularly valuable for resolving evolutionary histories at multiple taxonomic levels.

The beetle family Tenebrionidae, comprising over 20,000 species, includes numerous economically important pests and model organisms. Among them, *Latheticus oryzae* (Long‐headed flour beetle) is a notorious invasive pest of stored grains and fermented products, exhibiting remarkable adaptability to high‐temperature environments such as those in Chinese baijiu‐producing regions (Lv et al. 2025). With a slender body (2–3 mm) and distinctive protruding head morphology, *L. oryzae* infests wheat, rice, and fermented products like Daqu starter cultures, where both larvae and adults feed directly, causing significant yield losses. Despite its economic impact, the genomic resources for *L. oryzae* remain scarce, and its phylogenetic placement within Tenebrionidae remains contentious. Specifically, its hypothesized close relationship to the well‐studied genus *Tribolium* has never been robustly tested with genomic data, hindering a deeper understanding of its evolutionary ecology and the development of molecular management tools.

Although mitogenomes of related tenebrionids like *Tribolium castaneum* have been extensively characterized (Liu et al. 2016; Zhang et al. 2025), that of *L. oryzae* has not been available. Consequently, this study seeks to sequence and annotate the complete mitogenome of *L. oryzae* utilizing high‐throughput sequencing techniques. The research will characterize its structural features, including nucleotide composition, codon usage, and gene arrangement, and will perform phylogenetic analyses to elucidate its evolutionary relationships within the Tenebrionidae family. Additionally, the study will examine the expression patterns of mitochondrial protein‐coding genes (PCGs) to investigate functional adaptations associated with its pest ecology. This work aims to provide foundational data to inform mitogenome‐driven pest control strategies.

## 2 Materials and Methods

### 2.1 Insect Sample Collection and DNA Extraction

The *L. oryzae* specimens used in this study were collected in 2022 from infested sauce‐flavor Daqu in Moutai Town, Guizhou Province, China (27°51′ N, 106°22′ E). These beetles have been maintained on sauce‐flavor Daqu (temperature 30°C ± 1°C, humidity 60% ± 5%) in the laboratory since collection. Genomic DNA for mitogenome assembly was extracted from a pool of 30 adult *L. oryzae* individuals using the TIANamp Genomic DNA Kit (Tiangen, Beijing, China), following the manufacturer's instructions. The quality and concentration of the DNA samples was detected by a NanoDrop 2000 spectrophotometer and 1% agarose gel electrophoresis, respectively.

### 2.2 High‐Throughput Sequencing and Mitogenome Assembly

Whole genome shotgun (WGS) sequencing was performed on the Illumina NovaSeq platform. A library with 400 bp inserts was constructed and sequenced using paired‐end methodology at Personalbio (Nanjing, China). The raw sequencing data were first assessed for quality using FastQC v.0.11.9. Adapters were then removed using Trim Galore v.0.6.5, followed by a final quality validation with FastQC. High‐quality processed sequencing data were first assembled into Contigs using A5 miseq v20150522 (Coil et al. 2015), followed by Scaffold construction with SPAdes v3.9.0 (Bankevich et al. 2012). Gap filling between contigs was performed through collinearity analysis using Mummer v3.1 (Kurtz et al. 2004). Final mitochondrial sequence correction was completed using Pilon v1.18 (Walker et al. 2014).

### 2.3 Mitogenome Annotation and Analysis

The assembled mitogenome was annotated using the MITOS web server (http://mitos.bioinf.uni‐leipzig.de, accessed on March 2, 2025) (Bernt et al. 2013). The annotation results were confirmed by comparison with homologous sequences in the NCBI database, and the annotated genome was submitted to the NCBI. The mitogenome of *L. oryzae* was mapped using CGView (http://wishart.biology.ualberta.ca/cgview/, accessed on March 2, 2025). Base composition and relative synonymous codon usage (RSCU) of mtgenome were computed with PhyloSuite desktop platform (Zhang et al. 2020). The formulas AT skew = (A − T)/(A + T) and GC skew = (G − C)/(G + C) were used to calculate AT skew and GC skew, respectively.

### 2.4 The Spatiotemporal Expression Analysis of PCGs

We collected 30 early larvae (1 day old), late larvae (last instar larvae), early pupae (1 day old), late pupae (5 days old), and 1‐day‐old adults, respectively. For each developmental stage, 30 individuals were pooled to form one biological replicate, with three independent biological replicates per stage. All collected insects were immediately frozen in liquid nitrogen at −80°C for storage. Each group had three biological replicates. Furthermore, we collected 120 early larvae and 120 1‐day‐old adults. We dissected them to obtain head, epidermis, midgut, and fat body tissues. These tissues were also frozen in liquid nitrogen at −80°C. Each tissue sample had three biological replicates. Total RNA was extracted from all samples using the Trizol method. The RQ1 RNase‐Free DNase was used to remove genomic DNA. We measured the concentration and purity of the RNA using a spectrophotometer. The integrity of the RNA was checked using agarose gel electrophoresis. First‐strand cDNA was synthesized using the HiScript II 1st Strand cDNA Synthesis Kit (+gDNA wiper) (Vazyme Biotech Co. Ltd., Nanjing, China). The resulting cDNA was stored at −20°C for later use. We designed quantitative primers for the 13 PCGs using primer‐blast (NCBI) (Table A1). The *ribosomal protein S3* (*rps3*) gene was used as the internal reference gene for normalization. Although the stability of *rps3* was not empirically validated under our specific experimental conditions in *L. oryzae* , it has been demonstrated to be a reliable reference gene in closely related tenebrionid beetles, such as *T. castaneum* . The primers were synthesized by Tsingke Biotechnology (Chongqing, China). Quantitative real‐time PCR (qPCR) was performed using the Polarsignal SYBR Green qPCR mix (Mikx Biotech, Shenzhen, China) kit on an ABI 7500 PCR system (Applied Biosystems, USA). This was done to analyze the relative expression levels of the mitochondrial PCGs. Each sample was tested with three biological replicates and three technical replicates. We used the 2^−ΔΔCt^ method for quantitative data analysis.

### 2.5 Phylogenetic Analysis

Phylogenetic analyses were performed using DNA sequences from the newly sequenced mitogenome of *L. oryzae* and 35 other mitogenomes from GenBank, with *Mordellochroa milleri* and *Mordella atrata* as outgroups (Table 1). Phylogenetic analyses were performed in the software of PhyloSuite (Zhang et al. 2020). The dataset of 13 PCGs was used to construct phylogenetic trees. MAFFT (https://www.ebi.ac.uk/Tools/msa/mafft/, accessed on August 8, 2025) was used for DNA sequence alignment. Alignment gaps were removed with Gblocks. The sequence alignments were concatenated, and maximum likelihood (ML) phylogenetic trees were reconstructed using IQ‐TREE v2.2.0 (Nguyen et al. 2015). The Edge‐linked partition model was employed, as it facilitates linked branch length estimation across partitions, which is particularly suitable for mitochondrial genes that evolve as a single genetic unit. The analysis included 5000 ultrafast bootstrap replicates (Minh et al. 2013) and the Shimodaira–Hasegawa–like approximate likelihood‐ratio test (Guindon et al. 2010). The phylogenetic tree was visualized and enhanced using the iTOL online tool (https://itol.embl.de/, accessed on August 8, 2025).

**TABLE 1:** Mitogenomes of 34 Tenebrionidae species and two outgroup taxa used for phylogenetic analysis in this study.

ID	Organism	Family	Length (bp)
KX087267.1	*Cteniopus* sp.	Tenebrionidae	15,827
MT554375.1	*Cteniopinus ruber*	Tenebrionidae	16,010
MW802587.1	*Cteniopinus hypocrita*	Tenebrionidae	16,046
MW802586.1	*Borboresthes tibialis*	Tenebrionidae	15,377
MW802593.1	*Opatrum subaratum*	Tenebrionidae	15,828
MN745102.1	*Opatrum sabulosum*	Tenebrionidae	16,079
MW802592.1	*Heterotarsus carinula*	Tenebrionidae	16,861
KU236386.1	*Gonocephalum* sp.	Tenebrionidae	15,836
NC_025332.1	*Ulomoides dermestoides*	Tenebrionidae	15,434
KX087341.1	*Scaphidema metallicum*	Tenebrionidae	12,511
JX412842.1	*Platydema* sp. PLA01	Tenebrionidae	16,028
NC_041101.1	*Zophobas atratus*	Tenebrionidae	15,494
KT876915.1	*Uloma* sp.	Tenebrionidae	15,909
NC_026702.1	*Tribolium confusum*	Tenebrionidae	15,813
PV563855.1	*Tribolium castaneum*	Tenebrionidae	15,885
NC_024600.1	*Tribolium audax*	Tenebrionidae	15,925
NC_037196.1	*Tenebrio obscurus*	Tenebrionidae	15,771
NC_024633.1	*Tenebrio molitor*	Tenebrionidae	15,785
JX412808.1	*Paramarygmus* sp.	Tenebrionidae	15,884
KT876905.1	*Nalassus laevioctostriatus*	Tenebrionidae	15,529
PX331584.1	*Latheticus oryzae*	Tenebrionidae	15,798
MW802588.1	*Lagria rufipennis*	Tenebrionidae	15,465
MW802590.1	*Exostira schroederi*	Tenebrionidae	15,985
MW802589.1	*Chlorophila portschinski*	Tenebrionidae	15,833
MT548779.1	*Cerogria popularis*	Tenebrionidae	15,465
MH789725.1	*Amarygmini* sp.	Tenebrionidae	15,942
NC_049092.1	*Alphitobius diaperinus*	Tenebrionidae	15,511
NC_013554.1	*Adelium* sp. NCS‐2009	Tenebrionidae	16,449
NC_027256.1	*Asbolus verrucosus*	Tenebrionidae	15,828
JX412780.1	*Strongylium suspicax*	Tenebrionidae	12,561
MW802585.1	*Strongylium pinfaense*	Tenebrionidae	15,812
MW802591.1	*Strongylium nakanei*	Tenebrionidae	15,801
MW802584.1	*Strongylium kulzeri*	Tenebrionidae	15,808
MW201671.1	*Promethis valgipes*	Tenebrionidae	15,801
KX087318.1	*Mordellochroa milleri*	Mordellidae	15,760
NC_013254.1	*Mordella atrata*	Mordellidae	15,540

### 2.6 Statistical Analysis

The expression levels of the 13 PCGs were statistically analyzed and graphed using GraphPad Prism 8.0. Experiments were repeated three times independently, with data shown as mean ± standard deviation (SD). One‐way ANOVA was used for group comparisons, with *p* < 0.05 indicating significance.

## 3 Results

### 3.1 Mitogenome Composition

**FIGURE 1:**
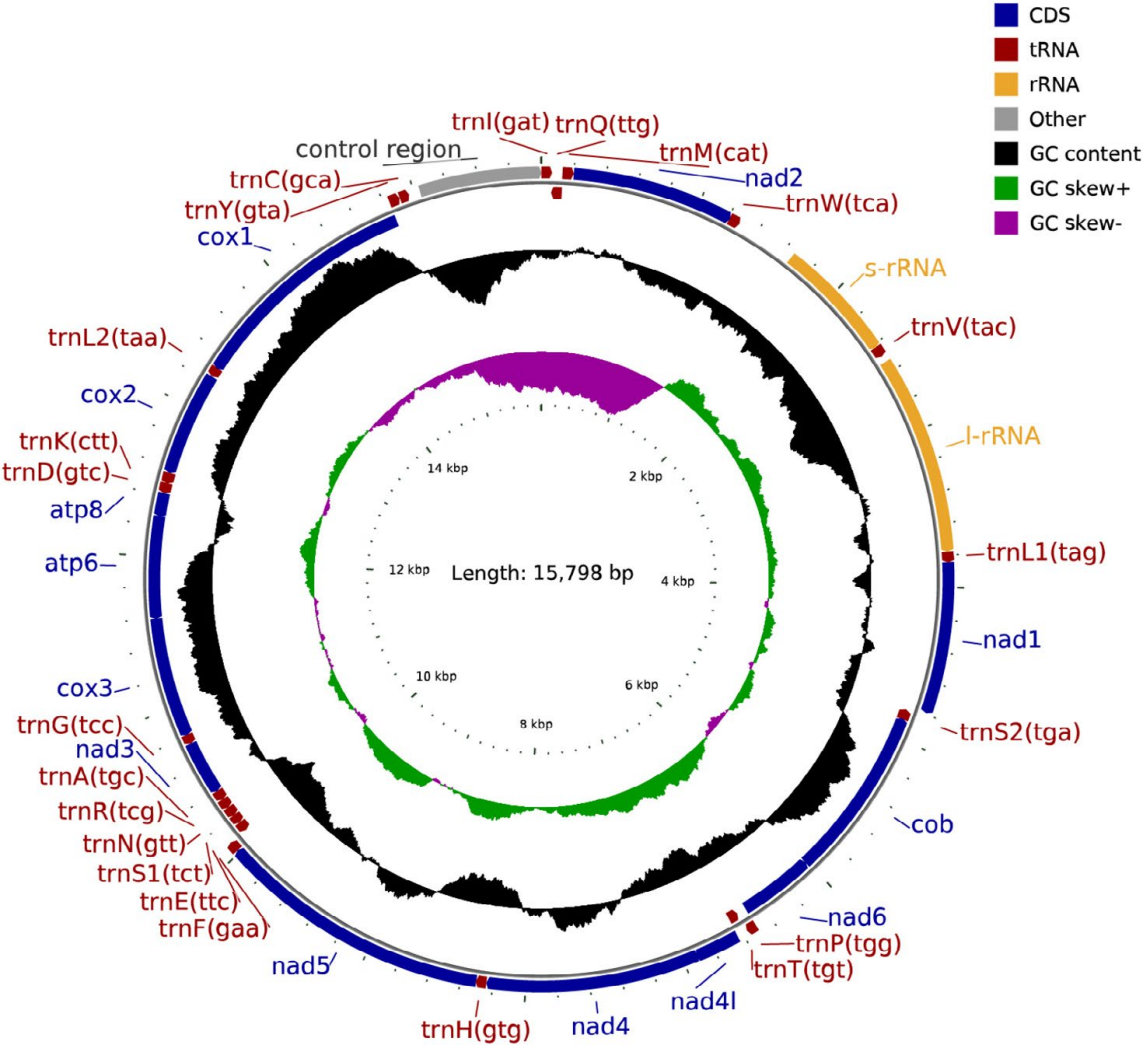
Mitogenome structure of *L. oryzae*.

The mitogenome of *L. oryzae* spans 15,798 base pairs (bp) and is deposited in GenBank accession number PX331584.1. It exhibits an adenine‐thymine (A + T) content of 71.08% and a guanine‐cytosine (G + C) content of 28.92% (Table 2). This genome comprises 13 PCGs, 22 tRNAs, 2 rRNAs (*rrnL* and *rrnS*), and a single noncoding control region (Figure 1). The J‐strand (minor strand, −) encodes 20 genes, including 8 PCGs and 12 tRNAs. In contrast, the N‐strand (major strand, +) encodes 17 genes, comprising 5 PCGs, 9 tRNAs, and 2 rRNAs (Table 2). The noncoding control region, situated between *trnC* and trnI, measures 762 bp in length and has an A + T content of 79.13% and a G + C content of 20.87% (Table 3). Within the coding region of the mitogenome of *L. oryzae* , 37 genes are present, featuring regions of overlapping genes and gene spacers between adjacent genes. There are 15 overlapping regions, ranging from 1 to 7 bp, with the longest overlap of 7 bp occurring between atp6 and *trnQ*, as well as between *nad4l* and *trn4*. Additionally, there are 10 gene spacers, varying from 1 to 381 bp, with the longest spacer of 381 bp located between *trnW* and *rrnS*. Furthermore, there are 12 regions that exhibit neither overlap nor spacer (Table 3).

**TABLE 2:** Nucleotide composition and skewness of the mitogenome of *L. oryzae* .

Region	A%	C%	G%	T%	A + T%	G + C%	AT_skew	GC_skew
Whole genome	31.35	10.97	17.95	39.73	71.08	28.92	−0.12	0.24
*rrnS*	31.63	7.97	17.12	43.27	74.90	25.10	−0.16	0.37
*rrnL*	30.60	8.03	17.03	44.34	74.94	25.06	−0.18	0.36
*nad2*	41.25	19.78	7.62	31.36	72.60	27.40	0.14	−0.44
*nad1*	20.72	9.57	18.82	50.89	71.61	28.39	−0.42	0.33
*cob*	33.6	21.64	12.23	32.54	66.14	33.86	0.02	−0.28
*nad6*	40.57	20.49	6.69	32.25	72.82	27.18	0.11	−0.51
*nad4l*	20.35	6.32	19.30	54.04	74.39	25.61	−0.45	0.51
*nad4*	22.68	9.04	19.97	48.30	70.99	29.01	−0.36	0.38
*nad5*	24.33	8.17	19.02	48.48	72.81	27.19	−0.33	0.40
*nad3*	31.36	24.29	9.60	34.75	66.10	33.90	−0.05	−0.43
*cox3*	31.00	21.47	13.98	33.55	64.55	35.45	−0.04	−0.21
*atp6*	32.59	22.27	10.76	34.38	66.97	33.03	−0.03	−0.35
*atp8*	41.03	19.87	5.13	33.97	75.00	25.00	0.09	−0.60
*cox2*	34.16	21.90	11.68	32.26	66.42	33.58	0.03	−0.30
*cox1*	30.60	21.55	14.65	33.20	63.80	36.20	−0.04	−0.19
Control Region	42.78	13.65	7.22	36.35	79.13	20.87	0.08	−0.31

**TABLE 3:** Annotation of the mitochondrial composition of *L. oryzae* .

Feature	Strand	Position (start‐end)	Length (bp)	Initiation_codon	Stop_codon	Anticodon	Intergenic_nucleotide
*trnI*	+	1–65	65			GAT	−3
*trnQ*	−	63–131	69			TTG	
*trnM*	+	132–200	69			CAT	
*nad2*	+	201–1211	1011	ATA	TAA		−2
*trnW*	+	1210–1278	69			TCA	381
*rrnS*	+	1660–2424	765				−1
*trnV*	+	2424–2492	69			TAC	38
*rrnL*	+	2531–3775	1245				
*trnL1*	+	3776–3840	65			TAG	
*nad1*	+	3841–4791	951	TTG	TAG		17
*trnS2*	−	4809–4875	67			TGA	−2
*cob*	−	4874–6010	1137	ATG	TAA		−1
*nad6*	−	6010–6502	493	AAT	A(AA)		1
*trnP*	+	6504–6568	65			TGG	
*trnT*	−	6569–6631	63			TGT	2
*nad4l*	+	6634–6918	285	ATG	TAA		−7
*nad4*	+	6912–8238	1327	ATG	T(AA)		3
*trnH*	+	8242–8305	64			GTG	
*nad5*	+	8306–10,019	1714	ATA	T(AA)		
*trnF*	+	10,020–10,084	65			GAA	−2
*trnE*	−	10,083–10,144	62			TTC	
*trnS1*	−	10,145–10,204	60			TCT	
*trnN*	−	10,205–10,268	64			GTT	−1
*trnR*	−	10,268–10,331	64			TCG	−1
*trnA*	−	10,331–10,396	66			TGC	−2
*nad3*	−	10,395–10,748	354	ATT	TAG		
*trnG*	−	10,749–10,811	63			TCC	−3
*cox3*	−	10,809–11,595	787	ATG	T(AA)		−1
*atp6*	−	11,595–12,263	669	ATA	TAA		−4
*atp8*	−	12,260–12,415	156	ATT	TAG		
*trnD*	−	12,416–12,481	66			GTC	−1
*trnK*	−	12,481–12,550	70			CTT	3
*cox2*	−	12,554–13,238	685	ATA	A(AA)		
*trnL2*	−	13,239–13,303	65			TAA	−5
*cox1*	−	13,299–14,834	1536	AAC	TAA		1
*trnY*	+	14,836–14,901	66			GTA	3
*trnC*	+	14,905–14,965	61			GCA	71
Control region	+	15,037–15,798	762				−1

*Note:* Negative values in the Intergenic_nucleotide column indicate overlapping regions between adjacent genes.

### 3.2 Protein‐Coding Genes and Codon Usage

The mitogenome of *L. oryzae* comprises 13 PCGs with a total length of 11,105 bp. Among these, the *nad5* gene is the longest, measuring 1714 bp, while the *atp8* gene is the shortest at 156 bp, located on the N and J strands, respectively. Ten genes utilize ATN as their start codon. Specifically, *nad5*, *nad2*, *cox2*, and *atp6* initiate with ATA; *nad4l*, *nad4*, *cox3*, and *cob* commence with ATG; *atp8* and *nad3* begin with ATT; *nad6* uses AAT; *cox1* employs AAC; and nad1 starts with TTG. The genes *nad5*, *nad4*, and *cox3* feature an incomplete termination codon represented by a single T, whereas *nad6* and *cox2* terminate with an incomplete codon of A. The remaining eight PCGs conclude with the complete termination codons TAA or TAG (Table 3).

**FIGURE 2:**
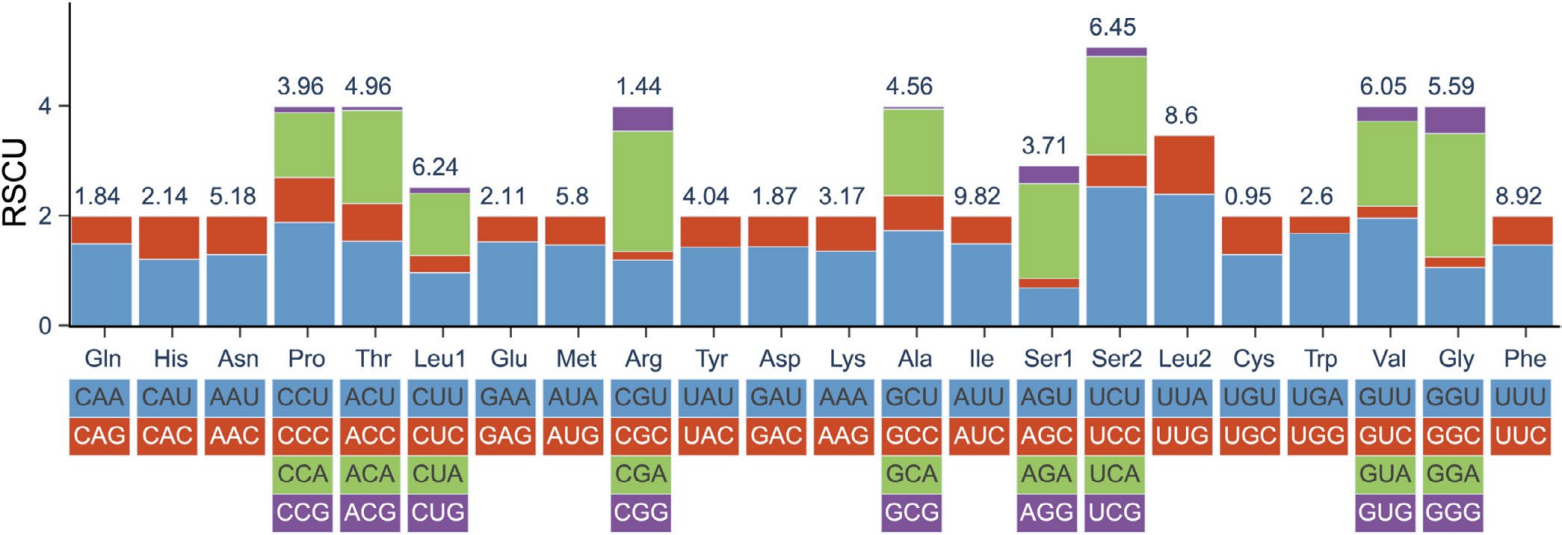
Relative synonymous codon usage (RSCU) in the mitogenome of *L. oryzae* . The box below the bar chart represents all codons encoding each amino acid, and the height of the column above represents the sum of all RSCU values. Values on the top of the bars denote amino acid usage.

The 13 PCGs of the *L. oryzae* mitogenome collectively encode 3705 codons, inclusive of stop codons. The most prevalent amino acids were isoleucine (Ile) at 9.82%, phenylalanine (Phe) at 8.92%, and leucine (Leu) at 8.60%, whereas cysteine (Cys) was the least common at 0.95% (Figure 2). Additionally, nearly all codons ending in adenine or uracil (A/U) demonstrated RSCU values exceeding one, with the exception of AGU, while codons ending in cytosine or guanine (C/G) generally exhibited RSCU values below one, except for UUG. The RSCU analysis highlighted significant variations in RSCU values among synonymous codons, suggesting a pronounced bias in codon usage (Figure 2).

### 3.3 Developmental and Tissue‐Specific Expression of PCGs

**FIGURE 3:**
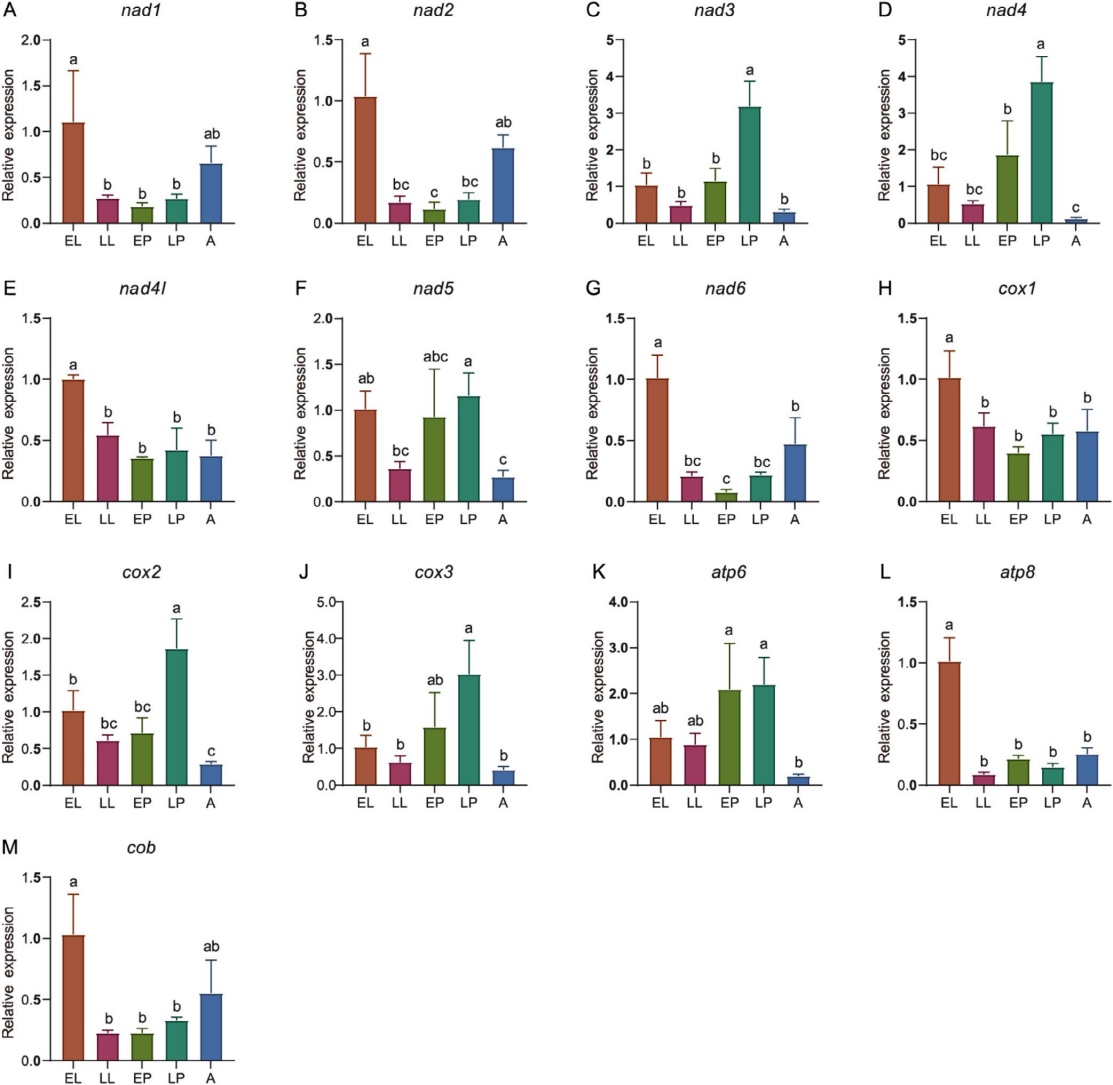
The expression patterns of mitochondrial protein‐coding genes at different developmental stages of *L. oryzae* . (A) *nad1*; (B) *nad2*; (C) *nad3*; (D) *nad4*; (E) *nad4l*; (F) *nad5*; (G) *nad6*; (H) *cox1*; (I) *cox2*; (J) *cox3*; (K) *atp6*; (L) *atp8*; (M) *cob*. A, adults; EL, early larvae; EP, early pupae; LL, late larvae; LP, late pupae. Expression levels were normalized to the EL stage. Error bars show standard deviation, and different lowercase letters above bars indicate statistically significant differences (Tukey's test, *p* < 0.05) between developmental stages.

The expression of the 13 mitochondrial PCGs varied significantly across developmental stages (Figure 3), reflecting shifting energy demands throughout the life cycle. Six genes encoding core subunits of complex I (*nad1*, *nad2*, *nad4l*, *nad6*) and the central electron transfer unit (*cox1*), along with *atp8*, were highly expressed in the early larval stage. This pattern suggests a heightened capacity for oxidative phosphorylation to meet the substantial energy requirements for rapid growth and development during this active feeding phase.

**FIGURE 4:**
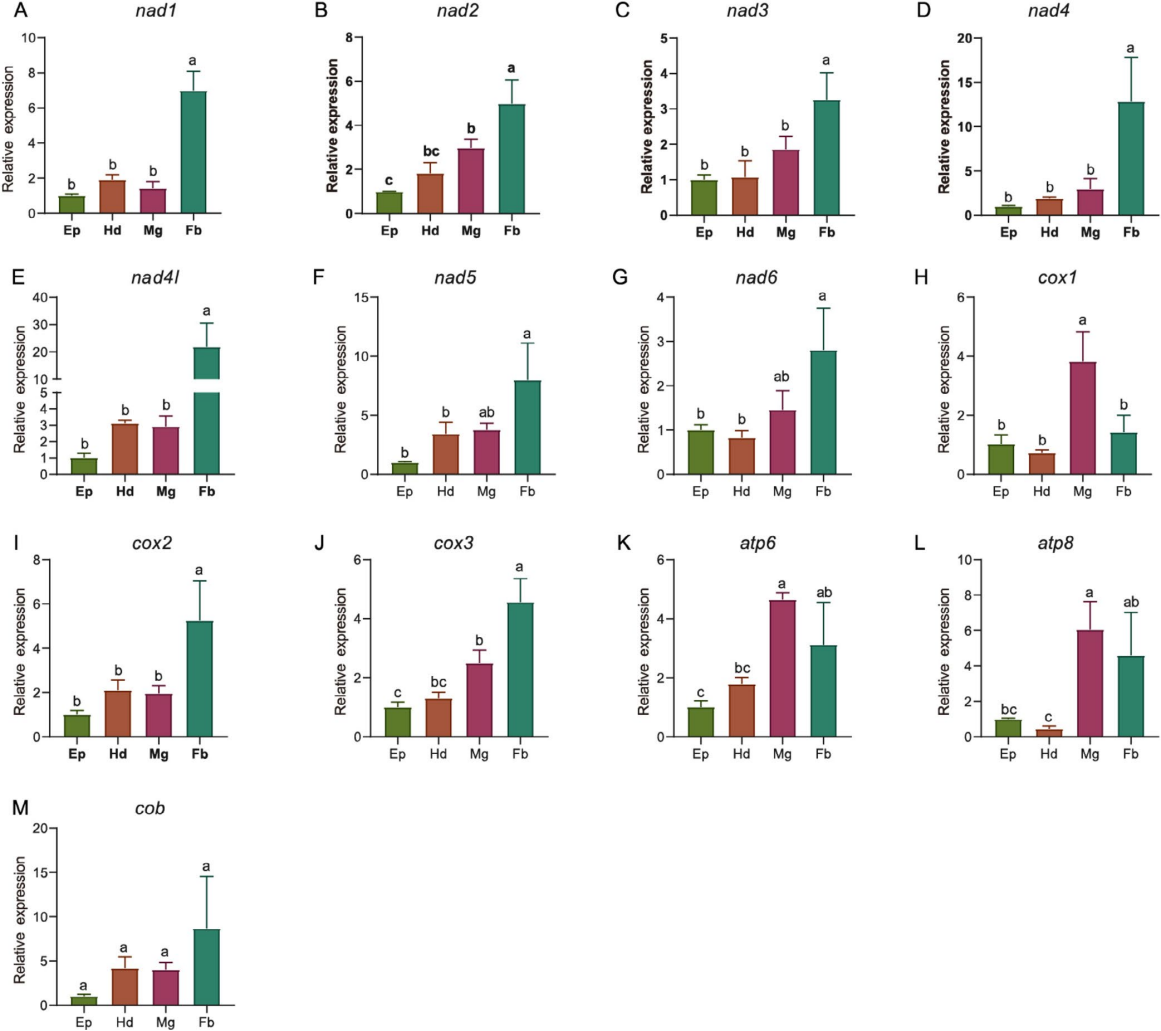
The expression patterns of mitochondrial protein‐coding genes at different tissues of *L. oryzae* early larvae. (A) *nad1*; (B) *nad2*; (C) *nad3*; (D) *nad4*; (E) *nad4l*; (F) *nad5*; (G) *nad6*; (H) *cox1*; (I) *cox2*; (J) *cox3*; (K) *atp6*; (L) *atp8*; (M) *cob*. Ep, epidermis; Fb, fat body; Hd, head; Mg, midgut. Expression levels were normalized to the Ep stage. Error bars show standard deviation, and different lowercase letters above bars indicate statistically significant differences (Tukey's test, *p* < 0.05) between developmental tissues.

To further investigate the spatial expression dynamics underlying these stage‐specific patterns, we analyzed PCG expression levels across four tissues in early larvae. The results revealed pronounced tissue‐specific expression (Figure 4). The majority of genes, including *nad1*, *nad2*, *nad3*, *nad4*, *nad4l*, *cox2*, and *cox3*, showed their highest expression levels in the fat body. As the primary organ for nutrient storage, lipid metabolism, and intermediary metabolism, the high mitochondrial metabolic activity in the fat body is consistent with its role in processing the abundant nutrients from the larval diet and converting them into stored energy and biosynthetic precursors for development. In contrast, *cox1* expression was significantly elevated in the midgut, potentially supporting the high energy demands of nutrient absorption and ion transport in this tissue.

### 3.4 tRNA and rRNA Structures

The mitogenome of *L. oryzae* comprises 22 tRNAs, collectively spanning 1437 base pairs and constituting 9.10% of the entire mitogenome. The individual tRNA genes range in length from 60 to 70 bp. Of these, 12 tRNA genes are located on the J‐strand, while 10 are situated on the N‐strand. Additionally, the J‐strand of the *L. oryzae* mitogenome harbors two rRNA genes, rrnS and *rrnL*, with lengths of 765 and 1245 base pairs, respectively. These rRNA genes are positioned within *trnW* and *trnV* (*rrnS*) and *trnV* and *trnL1* (*rrnL*).

### 3.5 Phylogenetic Analysis

**FIGURE 5:**
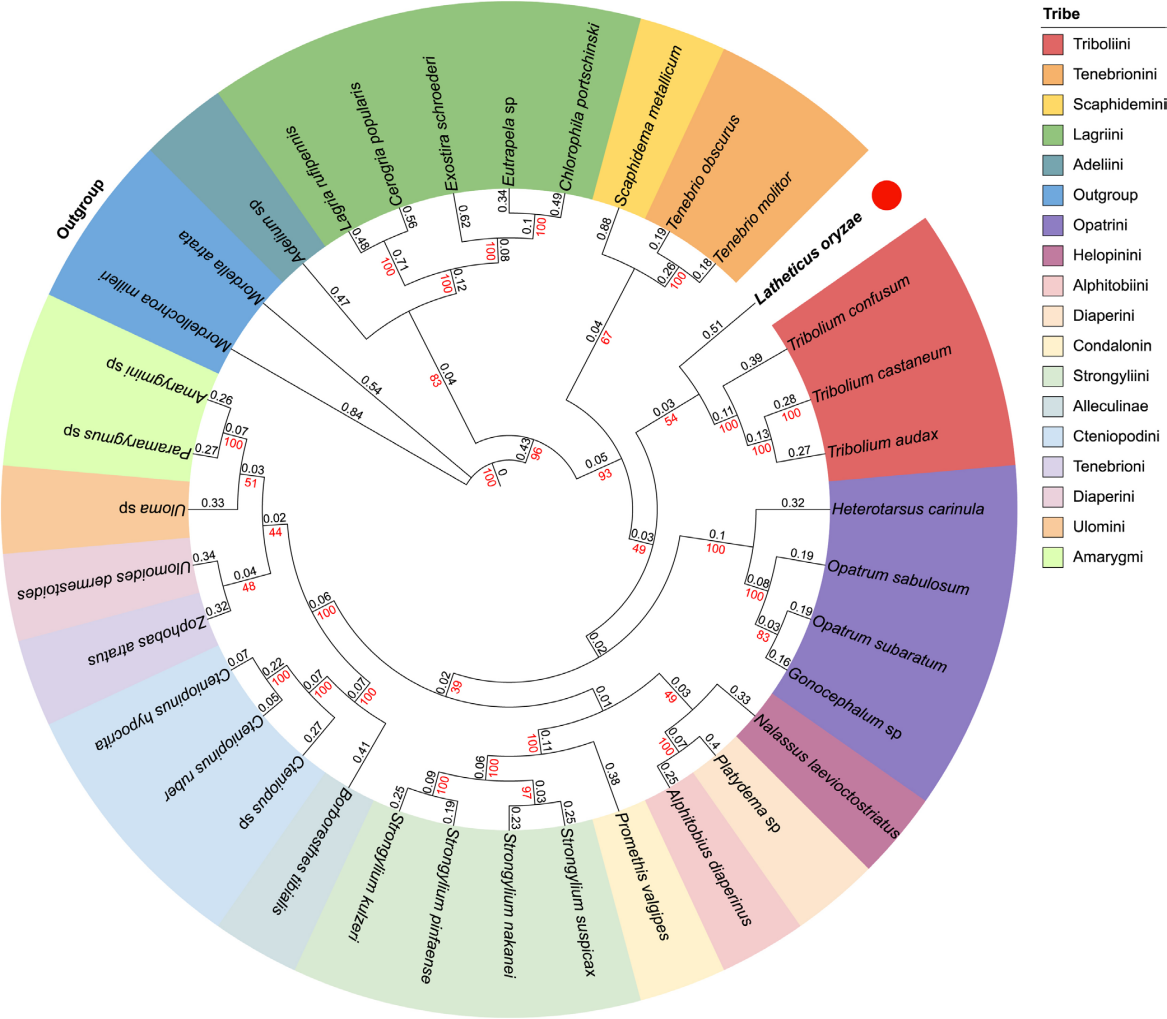
Phylogenetic relationships of the 34 Tenebrionidae species from maximum likelihood (ML) analysis based on 13 mitochondrial protein‐coding genes (PCGs). The branch length values (in black) and bootstrap support values (in red) are shown on corresponding nodes in the identical topology of tree. The different colors of species name blocks represent the different Tribes.

The ML phylogeny based on a concatenated dataset of 13 mitochondrial PCGs (36 taxa: 34 Tenebrionidae species, with *M. milleri* and *M. atrata* as outgroups) revealed that Tenebrionidae forms a strongly supported monophyletic clade (bootstrap = 100%), with the outgroups effectively anchoring the root of the tree. *L. oryzae* clustered tightly with *Tribolium* spp. with minimal genetic divergence. It showed greater genetic distance from other genera like *Tenebrio*, *Opatrum*, and *Gonocephalum*. Other Tenebrionidae species (e.g., *Strongylium* spp., *Zophobas atratus*) formed respective clades, reflecting diverse phylogenetic relationships (Figure 5).

## 4 Discussion

This study presents the first complete mitogenome of *L. oryzae* , providing new insights into the evolutionary history and adaptive biology of this widespread pest. The *L. oryzae* mitogenome exhibits architectural and compositional features typical of Coleoptera—including high A + T bias, conserved gene order, and variant initiation/termination codons—consistent with patterns observed across Tenebrionidae and other related families (Bai et al. 2018; Wei and Shi 2023; Wu et al. 2022). These characteristics not only reflect the structural constraints of insect mitogenomes but also underscore the role of mutational pressure and selection in shaping genomic evolution in the group.

The PCGs display a variety of start codons (ATA, ATG, ATT, AAT, AAC, TTG), which is a common feature in insect mitogenomes, including those of other Tenebrionid species (Liu et al. 2022; Wang et al. 2023). The use of non‐canonical start codons (e.g., AAC for *cox1*) has also been reported in Lepidoptera (Dai et al. 2015; Li et al. 2020; Yang et al. 2020) and Hymenoptera (Wang et al. 2022; Zhang et al. 2018), suggesting evolutionary flexibility in mitochondrial translation initiation. Termination codons include both complete (TAA, TAG) and incomplete (single T or A) forms, the latter of which are likely post‐transcriptionally polyadenylated to form functional stops—a widespread phenomenon in insect mitochondria (Xie et al. 2024).

Our analysis of mitochondrial PCG expression provides functional insights that align with the life history of *L. oryzae* as a stored product pest. The elevated expression of energy metabolism genes in larval stages—and their pronounced activity specifically within the fat body—points to a stage of intense mitochondrial biogenesis and oxidative phosphorylation. This is likely an adaptive response to support the rapid growth and nutrient accumulation required in the larval stage, where maximizing energy harvest from a nutrient‐rich but ephemeral resource is critical for fitness. The larval fat body acts as a metabolic hub, and the high density of mitochondria there is essential for converting dietary carbohydrates and lipids from grains and Daqu into stored energy and biomolecules for subsequent metamorphosis and reproduction. This efficient energy management system likely underlies the pest's success in resource‐rich storage environments. The tissue‐ and stage‐specific expression profiles further suggest functional partitioning of mitochondrial metabolism, potentially reflecting life‐history adaptations linked to the pest ecology of *L. oryzae* .

Our phylogenetic analysis firmly places *L. oryzae* within the tribe Tenebrionini as a sister group to *Tribolium*, resolving long‐standing uncertainty about its systematic position. This close relationship suggests possible shared ecological and evolutionary trajectories, including adaptation to stored grains and similar life‐history strategies. The *Tribolium*–*Latheticus* clade may represent a lineage that underwent rapid diversification in synanthropic environments, highlighting the role of ecological opportunity in the evolutionary radiation of tenebrionid beetles.

In summary, the *L. oryzae* mitogenome provides a valuable genomic resource for future studies on insect phylogenetics, molecular ecology, and pest evolution. The expression patterns and phylogenetic relationships revealed here deepen our understanding of how mitochondrial function may underlie ecological adaptations in stored‐product pests. Future work could integrate population genomics and functional assays to further explore the evolutionary mechanisms driving mitogenome diversity and adaptation in Tenebrionidae.

## Author Contributions

**Shan Xu:** data curation (equal), formal analysis (equal), investigation (equal), methodology (equal), software (equal), writing – original draft (lead). **Lihan Cheng:** data curation (equal). **Wennan Hu:** formal analysis (equal), validation (equal). **Yansong Gao:** formal analysis (equal), visualization (equal). **Qin Cheng:** conceptualization (lead). **Jun Lv:** project administration (equal), writing – review and editing (lead).

## Conflicts of Interest

The authors declare no conflicts of interest.

## Data Availability

The mitogenome sequence of *L. oryzae* generated in this study has been deposited in the GenBank database under the accession number PX331584 and can be downloaded via the link https://www.ncbi.nlm.nih.gov/nuccore/PX331584.1/.

## Appendix A

**TABLE A1:** Primer sequences used for RT‐qPCR in this study.

Primer name	Primer sequence (5′ to 3′)	Size (bp)
*atp6‐F*	TTTGCCCACCTTGTTCCACA	108
*atp6‐R*	TAGCCGAATGGCTAAGGTCC	
*atp8‐F*	AAATAGCACCGCTAAATT	121
*atp8‐R*	GTTGGGTATGATTTTTTGAA	
*cob‐F*	ACCCCTGTCCACATTCAACC	236
*cob‐R*	GGTCGTGCTCCAATTCAGGT	
*cox1‐F*	CATTCTTCGACCCTGCTGGA	257
*cox1‐R*	GGCTCGAGTGTCAACGTCTA	
*cox2‐F*	GCCACCCCTGGTCGATTAAA	133
*cox2‐R*	GGTTGGGAGCAATTCTTTCTGC	
*cox3‐F*	TTATGGCCACAGGATTCCACG	125
*cox3‐R*	GGCTGCTGCTTCAAATCCGA	
*nad1‐F*	TGCAGAAGGTGAGTCTGAACTG	208
*nad1‐R*	AGTTCCCCGAACCCAAATCC	
*nad2‐F*	AGCAGGATTACCCCCATTCA	253
*nad2‐R*	GGTGCATGCAGGTAGGCTTAT	
*nad3‐F*	CATCTGCACGTCTCCCGTT	197
*nad3R*	GGCTCCTTGCTTTCATTCGTG	
*nad4‐F*	TGTAGAGGCACCTGTTTCGG	276
*nad4‐R*	GACCCAGCTATACCCCACCT	
*nad4l‐F*	TCTTGACCTTTAGAGTGTGTGAGG	71
*nad4l‐R*	ACCATGTGTTCGAATTAAAGAAACT	
*nad5‐F*	GGGATGGTCTAGGTCTTGTCTC	111
*nad5‐R*	GAGCAACATCTCCAACCCGAT	
*nad6‐F*	TCATTTGCAACACAAAGAGAACT	152
*nad6‐R*	TGTCGAATTGAGCCTTGCTTG	
*rps3‐F*	ACTGACCGTGTTTTGGGAGA	189
*rps3‐R*	ACGACGAACAGCCAATCCTC	
